# Association between aminotransferase/alanine aminotransferase ratio and cardiovascular disease mortality in patients on peritoneal dialysis: a multi-center retrospective study

**DOI:** 10.1186/s12882-020-01840-7

**Published:** 2020-06-01

**Authors:** Xiaoran Feng, Yueqiang Wen, Fen Fen Peng, Niansong Wang, Xiaojiang Zhan, Xianfeng Wu

**Affiliations:** 1 Department of Nephrology, Jiujiang No. 1 People’s Hospital, Jiujiang, China; 2 Department of Nephrology, The Second Affiliated Hospital of Guangzhou Medical University, Guangzhou, China; 3 Department of Nephrology, Zhujiang Hospital of Southern Medical University, Guangzhou, China; 4 Department of Nephrology, Affiliated Sixth People’s Hospital, Shanghai Jiao Tong University, No.600, Yi Shan Road, Shanghai, China; 5 Department of Nephrology, The First Affiliated Hospital of Nanchang University, No. 17, Yongwaizheng Street, Donghu District, Nanchang City, 330006 Jiangxi province China

**Keywords:** Aspartate aminotransferase/alanine aminotransferase ratio, Cardiovascular disease, Mortality, Peritoneal dialysis

## Abstract

**Background:**

Elevated aspartate aminotransferase/alanine aminotransferase (AST/ALT) ratio is an independent risk factor for cardiovascular disease (CVD) among the general population. However, an association between AST/ALT ratio and CVD mortality in patients on peritoneal dialysis (PD) has received little attention.

**Methods:**

A total of 2224 incident PD patients from multi-centers were enrolled from November 1, 2005, to June 30, 2017, in this retrospective cohort study. The primary endpoint was CVD mortality. Eligible patients were divided into high and normal groups according to the AST/ALT ratio cut-off for CVD mortality with the receiver operating characteristic (ROC) curve. The associations between the AST/ALT ratio and CVD mortality were evaluated by the Cox regression model.

**Results:**

Of eligible 1579 patients with a mean age of 49.3 ± 14.6 years, 55.4% of patients were male, 18.1% of patients had diabetes, and 64.2% of patients had hypertension. The prevalence of a high AST/ALT ratio was 76.6% in the cohort population. During a follow-up period with 4659.6 patient-years, 316 patients died, of which 193 (61.1%) deaths were caused by CVD episodes. The incidence of CVD mortality in the high group was significantly higher than that in the normal group (13.1% versus 9.2%, *P* = 0.024). Cumulative CVD mortality rates were significantly different between the two groups by Kaplan-Meier analysis [hazards ratio (HR) = 1.50, 95% confidence index (CI) 1.09–2.07, *P* = 0.014]. After adjusting for confounding factors, a higher AST/ALT ratio was independently associated with an increased risk of CVD mortality compared with their counterparts (HR = 1.43, 95%CI 1.08–2.41, *P* = 0.002).

**Conclusions:**

PD patients with high baseline AST/ALT ratio levels may be at a significant risk of CVD mortality.

## Background

Cardiovascular disease (CVD) represents the leading cause of death in peritoneal dialysis (PD) patients, accounting for up to 40–60% of deaths [1, 2]. Traditional risk factors, such as diabetes, hypertension, dyslipidemia, and a history of CVD, account for up to 50% of CVD in dialysis patients. At the same time, renal specific markers, including anemia, disordered bone mineral metabolism, and oxidative stress, also likely contribute to the total CVD burden in these patients [3–7]. Therefore, exploring new non-traditional risk factors for CVD episodes may be beneficial to further improve the prognosis of PD patients.

Alanine aminotransferase (ALT) is only found in the liver, but aspartate aminotransferase (AST) in the liver and heart tissue [8]. Compared to ALT, AST significantly increased in patients with CVD events, presenting an elevated AST/ALT ratio of those patients [9, 10]. As compared to patients with a normal AST/ALT ratio, those with a high AST/ALT ratio had a higher pre-existing CVD rate, suggesting that the increased AST / ALT ratio may implicit heart load and injury and potential CVD episodes [11, 12]. Among the general population participating in a community-based health screening with a 10-year follow-up, the increased AST/ALT ratio had an independent association with CVD mortality, considering to be a new non-traditional risk for CVD episodes [8]. Another study from the United Kingdom showed that elevated AST/ALT ratio is significantly associated with an increased risk of developing CVD in men with no history of CVD at baseline [13]. It was noteworthy that patients on dialysis had reduced serum levels of aminotransferases [14, 15], whereas whether the AST/ALT ratio was an independent predictor of CVD mortality in dialysis patients remains unknown. In the present study, the aim of this study was to evaluate the association between the AST/ALT ratio and CVD mortality in PD patients.

## Methods

### Study design and population

A retrospective cohort study were conducted, with incident PD patients between November 1, 2005, and February 28, 2017, from four PD centers. Participants with aged less than 18 years at the start of PD or < three PD vintage were excluded by the end of study. The AST/ALT ratio can increase by alcohol consumption and cardio-hepatic interaction [16, 17]. Patients with pre-existing CVD or liver disease are at significant risk of CVD episodes. Thus, to minimize the effect of a history of CVD or liver disease on the association between AST/ALT ratio and CVD mortality, we excluded those with pre-existing CVD or liver disease. To further minimize selective bias, we excluded those with an AST/ALT ratio > 2, who may have underlying liver disease. Thus, patients were excluded from the study if they had current drinking, had been diagnosed with a history of CVD, chronic liver disease, or AST or AST values more than two times higher normal values. The study was approved by the Human Ethics Committee of each research center, consistent with the ethical principles of the Declaration of Helsinki. Eligible participants signed a informed consent.

Two experienced investigators at each center recorded demographic characteristics, laboratory variables, medical records and prescriptions at baseline, such as age, sex, body mass index (BMI) diabetes, hypertension, hyperlipidemia, gastrointestinal bleeding, Charlson comorbidity index (CCI), and current smoking. Ejection fraction, estimated glomerular filtration rate (eGFR), hemoglobin, serum albumin, AST, ALT, total bilirubin, cholesterol, triglycerides, high-density lipoprotein (HDL), low-density lipoprotein (LDL), high-sensitivity C-reactive protein (hs-CRP), N-terminal -prohormone BNP (NT-proBNP), and 24-h urine output at baseline were included.

CVD mortality and all-cause mortality were mainly interesting points. The lethal causes were evaluated by two experienced nephrologists at each center. Participants transferring to hemodialysis with less than three-month survival were thought not to be censored, indicating PD treatment failed. Participants were followed up until PD cessation, death, or May 31, 2017. Transferring to hemodialysis (for more than three months), transferring to other centers, renal transplantation, loss of follow-up, or still survival with a follow-up duration of 5 years were considered to be censored. Eligible participants were conducted PD schedules in the light of International Standardized Peritoneal Dialysis Guidelines.

#### Definitions

CVD is defined as coronary heart disease, arrhythmias, sudden death, congestive heart failure, or cerebrovascular disease [18]. Chronic liver disease is defined as hepatitis B or C, alcoholic and non-alcoholic liver disease, and autoimmune liver disease [13, 19, 20]. We defined aminotransferase elevation as any value above normal of ALT or AST based on a recent, nationally representative the United States survey (AST > 40 IU/L, or ALT > 43 IU/L) [21]. The comorbidity score was calculated using CCI [22]. eGFR was calculated using the Chronic Kidney Disease Epidemiology Collaboration equation [23].

### Statistical analysis

AST and ALT measured according to the standard measurements of Chinese. Data were expressed as mean ± standard deviation, percentages, or median (25th–75th percentile). All eligible patients were divided into high and normal groups according to the AST/ALT ratio cut-off for CVD mortality with the receiver operating characteristic (ROC) curve. Baseline variable comparisons between groups were conducted using *t-tests*, Mann-Whitney test, or χ^2^ analyses. The association between baseline variables and high AST/ALT ratio was analyzed using Logistic regression. Variables clinically considered to be associated with high AST/ALT ratio were picked into a multivariate-adjusted Logistic regression model. Survival analysis was undergone by the Kaplan-Meier curve, alongside with the log-rank test. The hazard ratio (HR) of the AST/ALT ratio for CVD and all-cause mortality were analyzed using Cox regression. Crude HR was first examined (Model 1), followed by adjusting for age, sex, CCI, current smoking, and medication use (Model 2). In the next Model 3, BMI, eGFR, hemoglobin, albumin, total bilirubin, cholesterol, triglycerides, hs-CRP, and 24-h urine output were enrolled to assess whether the AST/ALT ratio had an independent association with interesting points, independent of confounding factors. *P* < 0.05 was considered statistically significant. Statistical analysis was performed using GraphPad software 8.0 (GraphPad Prism Software Inc., San Diego, California) and R software package 3.6.0 (https://www.r-project.org/).

## Results

### Baseline characteristics

**Fig. 1:**
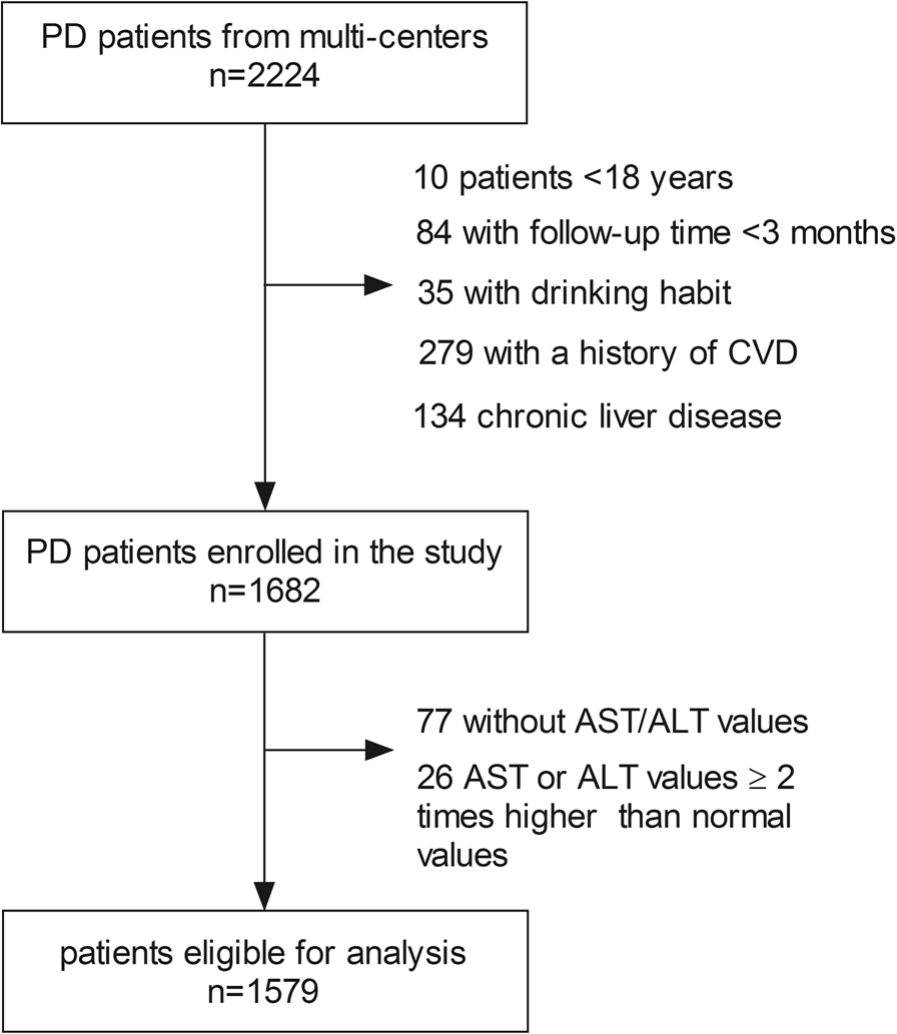
Patient flow in the study. PD, peritoneal dialysis; CVD, cardiovascular disease; AST/ALT, aspartate aminotransferase/alanine aminotransferase

A total of 2224 incident PD patients were enrolled in the present study, of whom ten patients younger than 18 years, 84 patients on PD < 3 months, 35 with current drinking, 279 with a history of CVD, 134 with chronic liver disease, 77 without baseline AST/ALT ratio, and 26 with AST or ALT values ≥two times higher than normal values were excluded. The remaining 1579 patients with baseline AST/ALT ratio were eligible for the present analysis (Fig. 1). Of 1579 patients with CCI of 3.87 ± 1.66, the mean age was 49.3 ± 14.6 years, 55.4% were male sex, 18.1% had diabetes, 64.2% had hypertension, and 16.2% had hyperlipidemia.

In ROC curve analysis (supplement), the AST/ALT ratio (area under curve = 0.75, 95% CI 0.72–0.77; *p* < 0.001) was found to be a significant predictor of CVD mortality with a sensitivity (79.4%) and specificity (75.8%). The cut-off of the AST/ALT ratio for CVD mortality was 1.0 in the cohort population. A total of 1210 (76.6%) patients were in the high group and 369 (23.4%) patients in the normal group. The baseline characteristics of the study population are shown in Table 1. Patients with high AST/ALT ratio were older (*P* < 0.001), likely to be female sex (P < 0.001), had higher frequency of hyperlipidemia (*P* = 0.029) and statin use (*P* = 0.037), had higher CCI (P < 0.001) and LDL (*P* = 0.038), and had a lower ALT values (*P* < 0.001) as compared to their counterparts.

**Table 1:** Baseline characteristics of patients stratified by baseline AST/ALT ratio

Variables	cohort (*n* = 1579)	Normal group (*n* = 369)	High group (*n* = 1210)	*P* value
Age (years)	49.3 ± 14.6	45.8 ± 13.7	50.3 ± 14.7	< 0.001
Male (%)	874 (55.4)	276 (74.8)	598 (49.4)	< 0.001
CCI	3.87 ± 1.66	3.59 ± 1.58	3.95 ± 1.68	< 0.001
Diabetes (%)	286 (18.1)	64 (17.3)	222 (18.3)	0.700
Hypertension (%)	1014 (64.2)	244 (66.1)	770 (63.6)	0.420
Hyperlipidemia (%)	256 (16.2)	46 (12.5)	210 (17.4)	0.029
Gastrointestinal bleeding (%)	41 (2.6)	10 (2.7)	31 (2.6)	0.853
Current smoking (%)	47 (3.0)	6 (1.6)	41 (3.4)	0.113
ACEI/ARB use (%)	518 (32.8)	114 (30.9)	404 (33.4)	0.410
Calcium antagonist use (%)	1141 (72.3)	272 (73.7)	869 (1.8)	0.507
β-blocker use (%)	505 (32.0)	122 (33.1)	383 (31.7)	0.611
Diuretic use (%)	94 (6.0)	20 (5.4)	74 (6.1)	0.707
Statin use (%)	157 (9.9)	26 (7.0)	131 (10.8)	0.037
BMI (kg/m^2^)	22.1 ± 3.6	22.3 ± 4.5	22.0 ± 3.3	0.305
Ejection fraction (%)	60.0 ± 5.9	59.8 ± 6.6	60.0 ± 5.7	0.820
eGFR (ml/min/1.73 m^2^)	5.63 (4.38–8.47)	5.81 (4.31–8.93)	5.50 (4.01–8.64)	0.367
Hemoglobin (g/dL)	8.6 ± 2.0	8.6 ± 2.1	8.6 ± 2.0	0.873
Albumin (g/dL)	3.5 ± 0.5	3.5 ± 0.5	3.4 ± 0.5	0.059
AST (IU/L)	18 (14–23)	19 (13–25)	18 (14–23)	0.086
ALT (IU/L)	13 (8–20)	36 (27–56)	8 (5–11)	< 0.001
Bilirubin (mg/dL)	0.30 (0.22–0.41)	0.33 (0.27–0.40)	0.30 (0.21–0.41)	0.565
Cholesterol (mg/dL)	159 ± 60	156 ± 55	160 ± 61	0.361
Triglyceride (mg/dL)	132 ± 97	126 ± 90	133 ± 98	0.198
HDL (mg/dL)	45.4 ± 16.3	44.7 ± 17.1	45.6 ± 16.1	0.432
LDL (mg/dL)	99.0 ± 38.8	95.1 ± 37.0	100.2 ± 39.3	0.038
hs-CRP (mg/L)	4.03 (1.97–11.04)	2.25 (0.85–19.5)	4.25 (2.04–14.20)	0.209
NT-pro-BNP (pg/mL)	2017 (800–6545)	771 (412–4835)	2375 (840–5760)	0.824
24-h urine output (mL)	851 ± 534	890 ± 589	839 ± 527	0.131

*AST/ALT* aspartate aminotransferase/alanine aminotransferase, *CCI* Charlson comorbidity index, *ACEI/ARB* angiotensin-converting enzyme inhibitor/angiotensin receptor blocker, *BMI* body mass index, *eGFR* estimated glomerular filtration rate, *HDL* high-density lipoprotein, *LDL* low-density lipoprotein, high-sensitivity C-reactive protein (*hs-CRP*), *NT-pro-BNP* N-terminal -prohormone BNP

### The high AST/ALT ratio

**Fig. 2:**
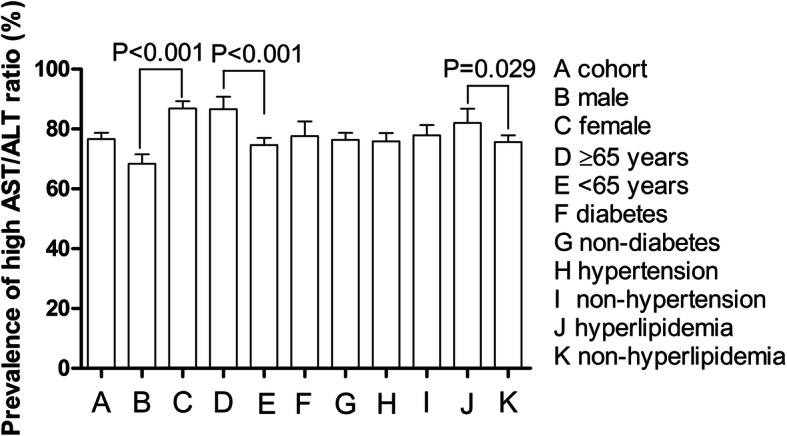
Prevalence of the high AST/ALT ratio in the cohort population and subgroups. AST/ALT, aspartate aminotransferase/alanine aminotransferase

The prevalence of the high AST/ALT ratio was 76.6% (74.5–78.7%) in the cohort population (Fig. 2). Multivariate Logistic analysis showed that older age [increased pre one year, HR = 1.02, 95% confidence interval (CI) 1.01–1.03, *P* < 0.001] and female (HR = 2.94, 95%CI 2.30–3.77, *P* < 0.001), statin using (HR = 1.66, 95%CI 1.22–2.44, *P* = 0.011), and lower bilirubin (HR = 0.54, 95%CI 0.33–0.90, *P* = 0.018) were independently associated with the high AST/ALT ratio (Table 2).

**Table 2:** Predictors for high AST/ALT ratio by Logistic regression

Variables	Multivariate Logistic regression	
	HR (95%CI)	*P* value
Age (increased pre 1 years)	1.02 (1.01–1.03)	< 0.001
Female (yes/no)	2.94 (2.30–3.77)	< 0.001
Current smoking (yes/no)	–	–
CCI (increased per 1 score)	–	–
Hypertension (yes/no)	–	–
Hyperlipidemia (yes/no)	–	–
Statin use (yes/no)	1.66 (1.12–2.44)	0.011
Bilirubin (increased per 1 mg/dL)	0.54 (0.33–0.90)	0.018

Variables clinically considered to be associated with high AST/ALT ratio were picked into a multivariate-adjusted Logistic regression model. *AST/ALT* aspartate aminotransferase/alanine aminotransferase, *CCI* Charlson comorbidity index, *LDL* low-density lipoprotein

### Baseline AST/ALT ratio and endpoints

**Fig. 3:**
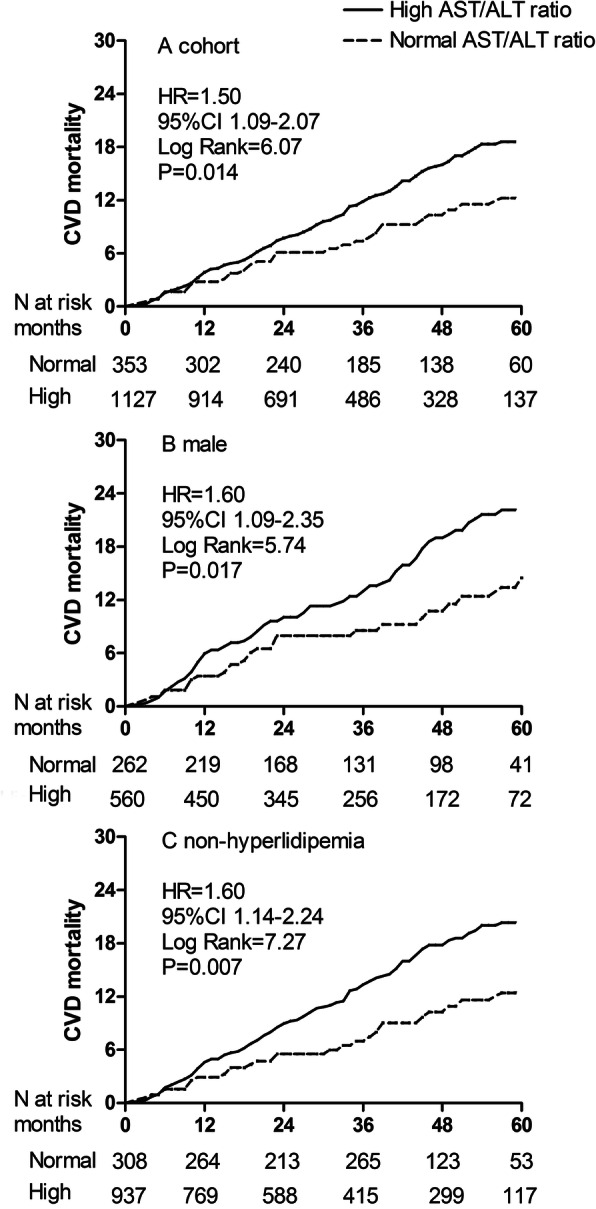
Cumulative CVD mortality curves in the cohort population and subgroups. **a** cohort; **b** male; **c** non-hyperlipidemia. AST/ALT, aspartate aminotransferase/alanine aminotransferase. CVD, cardiovascular disease

**Fig. 4:**
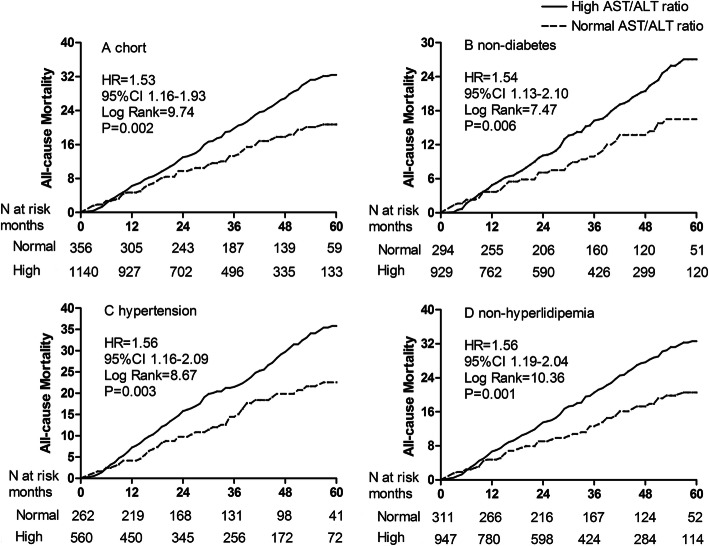
Cumulative all-cause mortality curves in the cohort population and subgroups. **a** cohort; **b** non-diabetes; **c** hypertension; **d** non-hyperlipidemia. AST/ALT, aspartate aminotransferase/alanine aminotransferase

The total follow-up period was 4659.6 patient-years. By the end of this study, 316 (20.0%) patients had died, 106 (6.7%) patients had undergone renal transplantation, 247 (15.6%) patients had transferred to hemodialysis, 18 (1.1%) patients had transferred to other PD centers, and 60 (3.8%) patients had been lost to follow-up; the remaining 832 (52.7%) patients were still followed at these PD centers. Of 316 deaths, 193 (61.1%) deaths were caused by CVD episodes. The Kaplan-Meier estimates showed that the cumulative CVD and all-cause mortality incidence were significantly different between two AST/ALT ratio groups (HR = 1.50, 95%CI 1.09–2.07, and HR = 1.53, 95%CI 1.16–1.93, Fig. 3a and Fig. 4a). At the end of 1, 3, and 5 years in this study, the incidence of CVD mortality was 8.1, 15.8, and 24.5% in the high group, and 6.1, 10.2, and 15.2% in the normal group, respectively. The incidence of all-cause mortality was 12.7, 26.7, and 32.6% in the normal group, and 9.8, 17.8, and 20.7% in the high group, respectively.

The association between the baseline AST/ALT ratio and CVD and all-cause mortality is shown in Table 3. Crude Cox model analysis showed that a high AST/ALT ratio was associated with an increased risk of CVD and all-cause mortality (HR = 1.63, 95% CI 1.13–2.27; HR = 1.58, 95%CI 1.18–2.10, Model 1). Multivariate Cox model analysis found that patients with a high AST/ALT ratio carried a higher risk of CVD and all-cause mortality (HR = 1.43, 95% CI 1.08–2.41, and HR = 1.45, 95% CI 1.13–2.37, Model 3), even after adjusting for confounding factors. In addition, the association between quartiles of AST/ALT ratio and CVD mortality were shown in Table 4.

**Table 3:** Adjusted hazards ratio for CVD and all-cause mortality using Cox regression models

	Model 1	Model 2	Model 3
	HR (95%)	HR (95%)	HR (95%)
CVD mortality	1.63 (1.13–2.27)	1.55 (1.10–2.36)	1.43 (1.08–2.41)
All-cause mortality	1.58 (1.18–2.10)	1.48 (1.16–2.24)	1.45 (1.13–2.37)

Hazards ratio: high AST/ALT ratio vs. normal AST/ALT ratio. Model 1: unadjusted. Model 2: adjusted for age, sex, CCI, smoking, and medication use. Model 3: model 2 adjusted for BMI, eGFR, hemoglobin, albumin, bilirubin, cholesterol, triglycerides, hs-CRP, and 24-h urine output

AST/*ALT* aspartate aminotransferase/alanine aminotransferase, *CVD* cardiovascular disease, *CCI* Charlson comorbidity index, *BMI* body mass index, *eGFR* estimated glomerular filtration rate, high-sensitivity C-reactive protein (*hs-CRP*)

**Table 4:** The association between quartiles of AST/ALT ratio and CVD mortality

	Model 1	Model 2	Model 3
	HR (95%)	HR (95%)	HR (95%)
Quartiles 1	Reference		
Quartiles 2	1.77 (1.24–2.51)	1.40 (0.96–2.05)	1.39 (0.96–2.03)
Quartiles 3	1.73 (1.20–2.50)	1.70 (1.20–2.41)	1.71 (1.21–2.43)
Quartiles 4	1.67 (1.16–2.41)	1.58 (1.10–2.27)	1.49 (1.20–2.18)

Model 1: unadjusted. Model 2: adjusted for age, sex, CCI, smoking, and medication use. Model 3: model 2 adjusted for BMI, eGFR, hemoglobin, albumin, bilirubin, cholesterol, triglycerides, hs-CRP, and 24-h urine output

*AST/ALT* aspartate aminotransferase/alanine aminotransferase, *CVD* cardiovascular disease, *CCI* Charlson comorbidity index, *BMI* body mass index, *eGFR* estimated glomerular filtration rate; high-sensitivity C-reactive protein (*hs-CRP*)

### Subgroup analyses

**Fig. 5:**
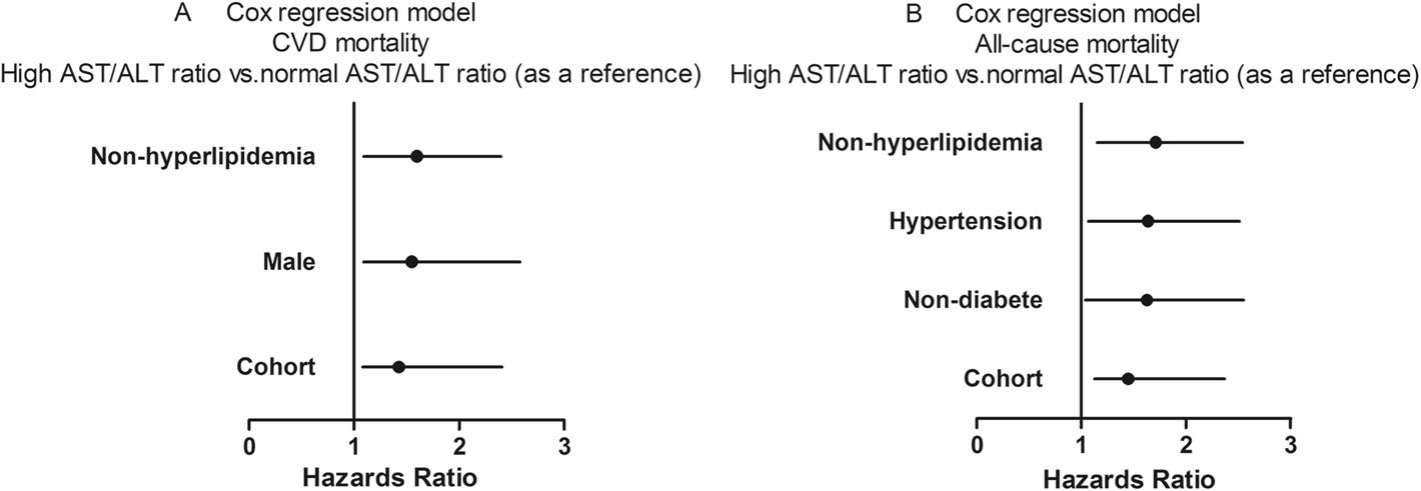
Adjusted HR for CVD and all-cause mortality with the Cox regression models. AST/ALT, aspartate aminotransferase/alanine aminotransferase. CVD, cardiovascular disease

The prevalence of high AST/ALT ratio ranged from 68.4% (95%CI 65.3–71.5%) to 86.9% (95%CI 84.3–89.3%) among all subgroups (Fig. 2). Survival analysis showed that the cumulative CVD mortality incidence between high and normal groups was a significant difference in the male and non-hyperlipidemia subgroups (Fig. 3 b and c). The cumulative all-cause mortality incidence between high and normal groups was a significant difference in the non-diabetes, hypertension, and non-hyperlipidemia subgroups (Fig. 4 b, c, and d). Adjusted HRs for CVD mortality were conducted in the male and non-hyperlipidemia subgroups, and for all-cause mortality in the non-diabetes, hypertension, and non-hyperlipidemia subgroups by the Cox regression models (Fig. 5).

## Discussion

In the present study, we found that higher baseline AST/ALT ratio may carry an increased risk of CVD and all-cause mortality in PD patients. Also, even though we excluded those patients with chronic liver disease, or a history of CVD, PD patients at the commencement of PD may have a higher prevalence of high AST/ALT ratio. PD patents have high risk of CVD episodes and liver disease, suggesting patients may have unknown underlying cardiovascular disease or liver disease. We should cautiously explain the association between AST/ALT ratio and CVD mortality.

Aminotransferase, including AST and ALT, is a well-known marker for liver injury. AST is in both the liver and myocardial tissue, but ALT is only in the liver [8]. The elevation of the AST/ALT ratio is due to induction by alcohol consumption and cardio-hepatic interaction [16, 24]. As compared to participants with a lower AST/ALT ratio, those with a high AST/ALT ratio had a higher pre-existing CVD prevalence, indicating a higher AST/ALT ratio may implicit heart damage and overload, as well as underlying CVD episodes [8]. The Japanese study of 3494 participants more than 40 years found that the high AST/ALT ratio had an independent association with all-cause and CVD mortality, with a 10-year follow-up [8]. In this study, participants with end-stage renal disease, incomplete data, or study withdrawal were excluded, but those with alcohol habits or pre-existing CVD were not excluded, suggesting less convincing of their findings due to selective bias. The Italy 6-year follow-up study with 2529 diabetes showed that the AST/ALT ratio had an independent association with all-cause and CVD mortality. Patients with a known history of drug-induced liver injury, viral hepatitis, cirrhosis of any etiology, and hemochromatosis were also excluded, but those with a history of CVD failed to be excluded in this study [25]. More recently, a 10-year follow-up prospective study of 29,316 participants aged between 25 and 84 years from the United Kingdom showed that an increased AST/ALT ratio were at significant risk of developing CVD episodes in male participants but not those female, with no baseline pre-existing CVD [13]. Nonetheless, when added to the traditional CVD prediction tools such as Framingham Risk Scores, the AST/ALT ratio did not contribute to any extra benefits in predicting the CVD accuracy. The major limitation was that patients with chronic liver disease were not excluded from this study. In the present study, to reduce selection bias, we excluded those current drinking, liver disease, and those with a history of CVD. We found that a higher AST/ALT ratio was independently associated with an increased risk for CVD and all-cause mortality. PD patients with a high AST/ALT ratio may have a 1.43-fold higher risk of CVD mortality and a 1.45-fold higher risk of all-cause mortality compared with their counterparts, even after adjustment for confounding factors. Subgroup analyses showed that a high AST/ALT ratio remained an independent predictor for CVD mortality in those male and non-hyperlipidemias, and all-cause mortality in those non-diabetes, hypertension, and non-hyperlipidemias. These findings suggested, along with previous studies, that PD patients with a higher AST/ALT ratio may have more CVD and all-cause involvement, and a preprocedural AST/ALT ratio, a widely available and inexpensive biomarker, might be helpful for risk stratification of CVD and all-cause mortality in PD patients.

A previous study reported that the prevalence of high AST/ALT ratio ≥ 1.0 was 37.9% in 2529 type 2 diabetes patients with a 6-year follow-up [13]. Patients with chronic liver diseases were excluded, but those with a history of CVD were not excluded from this study, which may lead to an over-estimated prevalence of high AST/ALT ratio. To date, the prevalence of the high AST/ALT ratio in dialysis patients has received little attention. In the present study, we excluded those with chronic liver disease or a history of CVD, which may be considered as an essential reason to increase the AST/ALT ratio. Nonetheless, the prevalence of a high AST/ALT ratio was 76.6% in the cohort study and ranged from 68.4 to 86.9% among all subgroups. Thus, there might be a higher prevalence of high AST/ALT ratio in PD patients. These findings suggested, along with previous studies, that future studies should further investigate the prevalence of the AST/ALT ratio in dialysis patients and whether the prognosis of PD patients might be improved by the management of the high AST/ALT ratio.

ALT has potential value as a novel biomarker of aging [13]. Decreased ALT resulted from a reduced liver size and liver blood flow and was associated with aging, frailty, and higher mortality in the general elderly population [26, 27]. There is a correlation between ALT levels and the severity of renal failure [15]. Patients on dialysis had reduced serum levels of aminotransferases, which suggested that the ALT levels were reduced concomitantly with the progression of renal dysfunction [14, 15]. Previous study demonstrated that ALT levels have a negative correlation with the severity of renal failure, resulting in high AST/ALT ratio in PD patients. In the present study, The ALT levels were reduced concomitantly with the progression of renal dysfunction, resulting in high prevalence of AST/ALT in PD patients. Thus, whether management of AST/ALT ratio can improve the prognosis of PD patients is worth further evaluation.

This study has some limitations. First, we retrospectively reported the independent association, but not causal relationships, between the AST/ALT ratio and the interesting points. We can not adjust all confounding factors of CVD and all-cause mortality, and not eliminated completely the residual confounding effect. Nonetheless, given the effect of residual confounding on the interesting outcomes, we adjusted for potential risk factors using multiply regression analysis. Second, PD patients usually took multiple drugs simultaneously due to other complications. So, it was difficult to determine which drugs may influence liver aminotransferase because of the interaction of drugs. Although we failed to exclude those patients whose liver aminotransferase may influence by multiple drugs, those with AST or ALT values ≥two times higher than normal values were excluded. Thus, the effect of drugs on liver aminotransferase may be minimized. Third, rare chronic diseases such as hemochromatosis, which may influence aminotransferase activity, failed to be excluded in the present study. Fourth, we only evaluated baseline variables rather than changes over time in these variables of CVD and all-cause mortality. Additionally, With the development of PD technique, medications, and the treatment of other complications, the prognosis of PD patients have been dramatically improved. Thus, changes over time may affect the results of PD patients, suggesting those receiving more advanced composited managements may gain more benefits than those ante-counterparts. Finally, because PD patients were all Chinese in the present study, the results may not apply to other ethnic PD patients.

## Conclusions

In conclusion, a high AST/ALT ratio at the initiation of PD was independently associated with an increased risk for CVD and all-cause mortality in PD patients. In addition, there may be a higher prevalence of high baseline AST/ALT ratio in PD patients. AST/ALT ratio, as a risk factor of non-traditional CVD mortality, should be monitored regularly in PD patients, reminding us of the evaluation of CVD episodes promptly. Fortunately, since laboratory assays for the AST/ALT ratio are common, readily available, and inexpensive, the AST/ALT ratio could be a promising parameter to identify PD patients at high risk for CVD and all-cause mortality. Future research should further investigate the prevalence of the AST/ALT ratio in PD patients and prospectively evaluate whether the prognosis of PD patients may be improved by the management of the AST/ALT ratio.

## Supplementary information

**Additional file 1.**

## Data Availability

The datasets used and/or analyzed during the current study are available from the corresponding author on reasonable request.

## Abbreviations

AST/ALT: Aspartate aminotransferase/alanine aminotransferase
CVD: Cardiovascular disease
CCI: Charlson comorbidity index
ACEI/ARB: Angiotensin-converting enzyme inhibitor/angiotensin receptor blocker
BMI: Body mass index
eGFR: Estimated glomerular filtration rate
HDL: High-density lipoprotein
LDL: Low-density lipoprotein
hs-CRP: High-sensitivity C-reactive protein
NT-pro-BNP: N-terminal -prohormone BNP
